# Exploring factors influencing the severity of pregnancy anemia in India: a study using proportional odds model

**DOI:** 10.1038/s41598-023-49872-x

**Published:** 2023-12-20

**Authors:** Iffat Ara Talin, Mahmudul Hasan Abid, Md Abdus Samad, Irma Domínguez Azpíroz, Isabel de la Torre Diez, Imran Ashraf, Abdullah-Al Nahid

**Affiliations:** 1 Electronics and Communication Engineering Discipline, Khulna University, Khulna, 9208 Bangladesh; 2 Department of Information and Communication Engineering, Yeungnam University, Gyeongsan-si, Gyeongsangbuk-do 38541 South Korea; 3 Universidad Europea del Atlántico, Isabel Torres 21, 39011 Santander, Spain; 4 Universidad Internacional Iberoamericana, 24560 Campeche, Mexico; 5 Universidade Internacional do Cuanza, Cuito, Bié Angola; 6 Department of Signal Theory, Communications and Telematics Engineering, Unviersity of Valladolid, Paseo de Belén, 15, 47011 Valladolid, Spain

**Keywords:** Computational biology and bioinformatics, Health care

## Abstract

Pregnancy-associated anemia is a significant health issue that poses negative consequences for both the mother and the developing fetus. This study explores the triggering factors of anemia among pregnant females in India, utilizing data from the Demographic and Health Survey 2019–21. Chi-squared and gamma tests were conducted to find out the relationship between anemia and various socioeconomic and sociodemographic elements. Furthermore, ordinal logistic regression and multinomial logistic regression were used to gain deeper insight into the factors that affect anemia among pregnant women in India. According to these findings, anemia affects about 50% of pregnant women in India. Anemia is significantly associated with various factors such as geographical location, level of education, and wealth index. The results of our study indicate that enhancing education and socioeconomic status may serve as viable approaches for mitigating the prevalence of anemia disease developed in pregnant females in India. Employing both Ordinal and Multinominal logistic regression provides a more comprehensive understanding of the risk factors associated with anemia, enabling the development of targeted interventions to prevent and manage this health condition. This paper aims to enhance the efficacy of anemia prevention and management strategies for pregnant women in India by offering an in-depth understanding of the causative factors of anemia.

## Introduction

Anemia is a global health issue affecting pregnant women and is a leading cause of maternal morbidity and mortality^1,2^. Anemia is characterized by a hemoglobin (Hb) level lower than 11 g/dL^3^. In India, pregnancy-associated anemia is a significant public health concern^4,5^. Pregnancy-associated anemia can be caused by inadequate dietary intake, insufficient iron consumption, or pre-existing conditions^6–8^. In India, an alarming proportion of pregnant women suffer from anemia, which makes it a major public health concern^4,5^. The Indian government has taken various measures to prevent anemia in pregnant women. Examples include distributing iron and folic acid supplements and conducting nutrition education programs^9,10^. Despite the preventive measures mentioned earlier, anemia remains a common problem among pregnant females in India. It is therefore necessary to investigate the factors that contribute to anemia in India.

The Demographic and Health Surveys (DHS) program^11^ collects data on population, health, and nutrition in more than 90 nations, including India. DHS also provides data on a range of health indicators, such as anemia, tuberculosis (TB), and high blood pressure (BP). Processing the DHS data can help determine the factors correlated with anemia within this particular demographic. This study aims to examine two points regarding anemia through processing the DHS dataset—firstly, to analyze the prevalence of anemia among pregnant women in India and secondly, to discover the factors that are associated with it. In order to assess the correlation between anemia and various factors such as geographic region, place of residence, education level, the origin of drinking water, socioeconomic status, religion, iodine intake, toilet facility, body mass index (BMI), and age, we employ the Chi-square test and gamma test. Furthermore, we used multinominal logistic regression to develop a comprehensive understanding of the influencing factors of anemia in India^12–14^. To develop a deeper understanding of the relationship between socioeconomic and sociodemographic factors and anemia, both ordinal logistic regression and multi-nominal logistic regression analysis have been used in this study. Our research aims to enhance the comprehension of the primary determinants of anemia during pregnancy in India for policymakers, healthcare providers, and researchers. The aim is to identify the principal causal factors of anemia in order to facilitate the creation of focused interventions that can alleviate the impact of this significant public health concern.

### Literature review

Anemia is a prevalent public health issue among pregnant women globally, with a higher incidence in developing nations like India. There has been much research conducted on the factors contributing to anemia during pregnancy in India, focusing on demographics, socioeconomics, nutrition, and health issues. The findings of research conducted in Jhalawar, Rajasthan, India^15^ reveal a substantial prevalence of anemia (81.1%) among pregnant females in a rural locality. The study also identifies certain sociodemographic variables that correlate significantly with anemia. The findings underscore the need for interventions to improve access to nutritional resources and antenatal care services to address this issue. Similarly, another study conducted in Karnataka, India, found that anemia was significantly associated with lower education levels and a lower household income^16^. Besides demographic and socioeconomic variables, numerous research studies have explored the correlation between prenatal anemia and dietary adequacy^17^ discusses the prevalence of anemia among pregnant women and adolescent females in India. The results show that almost (85%) of pregnant women and over (58%) of adolescent females are anemic. The lack of anemia prevention and control programs for adolescent girls is a significant problem. The study recommends implementing effective programs, especially for adolescent girls who do not receive iron and folic acid supplementation and have low serum ferritin levels. Insufficient intake of folic acid and vitamin B12 are additional nutritional factors recognized as potential risk factors for anemia during pregnancy in India^18^. Several studies have also investigated the association between anemia during pregnancy and health-related factors such as infections and chronic diseases. In a study conducted in a rural region of Haryana, India, parasitic infestations were found to be substantially linked to anemia during pregnancy^19^. Another study carried out in a tertiary care hospital in Mumbai, India, found that women with sickle cell anemia were at higher risk of anemia during pregnancy^20^.

While previous studies have identified various factors associated with anemia during pregnancy in India, there is limited research on the relative importance of these factors and their combined effects on anemia prevalence. For example^21^, found that several risk factors were significantly associated with LBW neonates, including anemia, pregnancy-induced hypertension, pre-pregnancy maternal weight less than 45 kgs, maternal height of less than 145 cm, and inadequate antenatal care. In addition, a large number of rural mothers did not receive antenatal care services or did not use them adequately, which indicated the need for health education, socioeconomic development, maternal nutrition, and increased access to health services during pregnancy. Another study^22^ reported that maternal education, socioeconomic status, and maternal body mass index directly correlate to birth weight. Underprivileged mothers have a higher prevalence of anemia and give birth to a greater number of very premature babies, they found. The study^23^ investigates the pregnant women’s risk factors linked to anemia during pregnancy through a prospective-retrospective cohort study of antenatal and postnatal women. According to the study, the prevalence of anemia was high, particularly among women with low socioeconomic status. The study also identified several other factors that contributed to anemia, including education, age, spacing, parity, history of bleeding, worm infestation, knowledge regarding anemia during pregnancy, gestation period, ability to choose food, and compliance with iron supplementation.

A study conducted in South India examined the relationship, between mental health and anemia during pregnancy. The findings revealed that women with levels of stress and anxiety were more susceptible to anemia. This suggests that interventions addressing pregnancy-related anemia should consider health aspects well^24^. Another research conducted in the slums of Kolkata, India highlighted the role of factors like poor sanitation and water quality in contributing to the prevalence of anemia among pregnant women^25^. To understand the impact of cultural practices on anemia prevalence, a study was conducted in Odisha, India. The study discovered that traditional beliefs and taboos related to foods during pregnancy led to deficiencies. This emphasizes the importance of interventions that are culturally sensitive^26^. Additionally, a study in Mumbai, India explored how maternal employment influenced anemia rates. It found that working mothers faced challenges in accessing care indicating the need for policies supporting maternal health within the workforce^27^.

Although the previous studies provided significant insights into the occurrence and underlying causes of anemia among pregnant women in India, further research is necessary to identify the key determinants contributing to anemia in this population. Moreover, most studies conducted in India have utilized simple logistic regression, which may not account for the ordinal nature of the severity of pregnancy anemia. In this study, ordinal logistic regression is used to determine the primary determinants of anemia among pregnant women in India which provides us with a better understanding of the risk factors that are associated with it. Furthermore, our analysis utilizes the latest DHS dataset, and to the best of our knowledge, the IDHS 2019–21 dataset has not been previously analyzed to assess the prevalence of pregnancy anemia in India.

### Assumptions

In conducting this study on the factors that affect the severity of pregnancy anemia in India using an odds model, we have some assumptions. Firstly, we trust that the DHS 2019–21 dataset accurately and reliably captures all the variables related to pregnancy anemia, including socioeconomic, nutritional, and health-related information. Moreover, we assume that the sample selected from this dataset represents the population of women in India and reflects their diverse geographic, cultural, and economic backgrounds. Our study assumes that utilizing techniques like Chi-squared tests, gamma tests as well as ordinal and multinomial regression is both appropriate and valid for analyzing the connections between identified factors and the severity of pregnancy anemia, in an Indian context. Finally, we want to acknowledge that our study is observational in nature and we should not assume that the relationships we found between factors and the severity of pregnancy anemia in the odds model imply causation. This highlights the fact that this research is exploratory and focused on correlations, then establishing cause and effect relationships.

### Organization of the paper

Firstly, we present an overview of anemia and its effects on maternal and fetal health. The present study investigates the existing literature regarding the prevalence and causative factors of anemia that develop in females during pregnancy in India. In order to explicate our research methodology, we expound upon the data and methods employed in our analysis in the Materials and Methods section. The Chi-squared and gamma tests were employed to evaluate the correlation between anemia and sociodemographic and economic variables. In contrast, ordinal and multinominal logistic regression was utilized to identify the most significant determinants of anemia. In the later section, the limitations of our study and its future aspects have been discussed. In the Results section, we present the results of our analyses, which are then discussed in the Discussion section. Lastly, we draw our paper to a close in the Conclusion section, summarizing our main findings.

## Materials and methods

### Data sources

The primary data for this study is obtained from the Demographic and Health Survey (DHS) dataset, which was conducted in the years 2019 to 2021. The DHS is a nationally representative survey conducted in numerous countries in the world including India. The aim of this survey is to collect key information about public health, demographics, and socio-economic factors countrywide. The data set is available on DSH’s website^11^ upon request and does not contain any identifiable information about the study participants. The selection of the DHS 2019–2021 dataset is intended to offer a current perspective on the factors that contribute to the severity of pregnancy anemia, in India. The DHS follows a nationally representative sampling procedure, ensuring the inclusion of diverse demographics. Additionally, DHS measures anthropometric indicators objectively and collects a variety of monitoring and impact assessment statistics. The survey also boasts a high response rate, further enhancing the reliability and validity of the gathered data. A total of 27494 respondents were included in this study after limiting the data selection to pregnant women and eliminating any missing valued samples.

### Variables

In this research, we examined the anemia status of pregnant women, which was initially categorized as severe, moderate, mild, and not anemic. To enhance the statistical analysis, we merged the ‘severe’ and ‘moderate’ as ‘severe to moderate’, and recategorized the target variable into three groups: severe to moderate, mild, and not anemic. We used explanatory variables at both the individual and household levels to conduct our study, such as type of residence, source of drinking water, type of toilet facility, sex of household head, wealth index, the religious affiliation of household head, educational level, geographic region, iodine level in salt, and high blood pressure, BMI, and age^12–14^. The variables “Wealth index” and “Educational level” have ordinal categories, while the others have nominal categories. The categories for each variable and the corresponding anemic status are provided in Table 1. Latrines with flushing to the piped sewer system, flushing to the septic tank, flushing to pit latrine, Ventilated improved pit latrine, Pit latrine with slab and Composting toilets are considered as safe toilet systems and others are unsafe^28,29^. Similarly, Piped into dwelling, Piped to the yard, Public tap or standpipe, Tube well or borehole, Protected dug well, Protected spring, Rainwater, and Bottled water are considered safe water sources. All other types of water sources are considered unsafe water sources^29,30^.

### Ordinal logistic regression

Ordinal logistic regression is a statistical method used to model the relationship between an ordinal outcome variable and one or more predictor variables^31,32^. Unlike binary logistic regression, which is used for binary outcomes, ordinal logistic regression is used for outcomes that have three or more ordered categories. In this model, the probability of an outcome falling into a particular category is transformed into a logit value that is linearly related to the predictor variables. The cumulative logits function can be written as^33,34^:

**Table 1:** Description of the dataset.

Variables	Measurement scale	Categories	Anemia level			Std. error	95% C.I.	
			Severe and moderate (%)	Mild (%)	Not anemic (%)		L.B.	U.B.
Place of residence	Nominal	Urban	22.0	21.8	56.2	0.002	1.803	1.812
		Rural	27.8	24.3	47.9			
Sex of household head	Nominal	Male	26.6	23.5	49.9	0.002	1.144	1.153
		Female	27.1	25.8	47.2			
Wealth index	Ordinal	Poorest	32.9	26.2	40.9	0.008	2.713	2.745
		Poorer	27.5	24.4	48.1			
		Middle	25.4	23.1	51.5			
		Richer	24.0	22.6	53.4			
		Richest	19.4	21.3	59.4			
Highest educational level	Ordinal	No education, preschool	34.4	24.9	40.7	0.006	1.719	1.740
		Primary	30.2	24.4	45.4			
		Secondary	25.9	24.0	50.1			
		Higher	19.2	21.5	59.3			
Result of salt test	Nominal	No iodine	28.2	24.8	47.0	0.001	0.943	0.948
		Iodine present	26.6	23.8	49.7			
High BP	Nominal	No	26.6	23.8	49.6	0.001	0.057	0.063
		Yes	26.7	23.8	49.5			
Type of toilet facility	Nominal	Improved	24.6	23.1	52.3	0.005	1.445	1.464
		Unimproved	28.4	23.9	47.7			
		No facility	33.7	26.4	39.9			
Source of drinking water	Nominal	Improved	26.7	23.8	49.4	0.002	1.116	1.123
		Unimproved	26.0	23.7	50.4			
Geographic region	Nominal	Northern	25.4	21.7	52.8	0.010	3.008	3.049
		Western	28.5	23.3	48.3			
		Eastern	33.4	28.6	38.0			
		Northeastern	20.3	20.8	58.9			
		Central	27.5	26.2	46.3			
		Southern	24.5	24.0	51.6			
Religion	Nominal	Hindu	28.1	24.7	47.2	0.116	5.180	5.633
		Muslim	25.3	23.3	51.5			
		Christian	17.1	19.1	63.8			
		Other/no religion	27.8	20.9	51.3			
BMI	Nominal	18.5 (underweight)	29.3	26.1	44.6	0.004	2.129	2.145
		18.5 to 24.9 (Normal weight)	27.1	23.9	49.1			
		25 to 29.9 (Overweight)	23.9	22.2	53.8			
		30 or more (Obese)	23.7	23.4	52.9			
Age	Nominal	< = 20	28.9	25.9	45.1	0.003	1.943	1.956
		21–30	26.4	23.7	49.9			
		31–40	24.5	21.5	54.0			
		40+	31.5	15.4	53.1			

1 $$\begin{aligned} \textrm{logit}(P(Y \le j)) = \beta _{j0} + \beta _{j1}x_1 + \beta _{j2}x_2 + \cdots + \beta _{j_p}x_p \end{aligned}$$

where $P(Y \le j)$ indicates the probability of the outcome variable being less than or equal to category *j*, $\beta _{j0}$ represents the intercept of category *j* and $\beta _{j1}$ to $\beta _{j_p}$ correspond to the coefficients associated with the predictor variables $x_1$ to $x_p$, respectively.

It can be seen from equation 1 that there is a correlation between the predictor variables and the likelihood of the outcome variable falling into each category. For each category j, the coefficients represent the difference in log odds between category j and category j-1, and the model takes these differences as constant across all predictor variables. Ordinal logistic regression is used in many fields of science, such as psychology, medicine, and social sciences. Specifically, it is used where the researchers are interested in predicting outcomes that have multiple ordered categories. It is possible to use both categorical and continuous predictor variables in the model, and maximum likelihood estimation is typically used in its implementation.

### Multinomial logistic regression

Multinomial Logistic Regression is a technique that helps us model and analyze the relationship, between categorical outcomes and one or more predictor variables^35,36^. It is an expansion of binary logistic regression designed to handle scenarios where the dependent variable has more than two categories. In this approach, we create logistic regression models for each category of the dependent variable comparing them to a reference category. We calculate probabilities for each category. Predict the one with the highest probability. Assuming *Y* represents the categorical dependent variable with *K* categories, and *X* denotes the predictor variables, the formula for regression can be expressed as follows^37,38^:

$$\begin{aligned} P(Y = k|X) = \frac{e^{\beta _{0k} + \beta _{1k}X_1 + \beta _{2k}X_2 + \ldots + \beta _{pk}X_p}}{1 + e^{\beta _{01} + \beta _{11}X_1 + \beta _{21}X_2 + \ldots + \beta _{p1}X_p} + e^{\beta _{02} + \beta _{12}X_1 + \beta _{22}X_2 + \ldots + \beta _{p2}X_p} + \ldots + e^{\beta _{0K} + \beta _{1K}X_1 + \beta _{2K}X_2 + \ldots + \beta _{pK}X_p}} \end{aligned}$$

where the variable $P(Y = k|X)$ represents the likelihood of the variable *Y* belonging to category *k*. The coefficients, for each predictor variable, for category $k$ are represented by $\beta _{0k}, \beta _{1k}, \beta _{2k}, \ldots , \beta _{pk}$. $X_1, X_2, \ldots , X_p$. $X_1, X_2, \ldots , X_p$ are the predictor variables. And the total number of categories in the dependent variable is represented by $K$.

### Chi-squared test

The chi-squared analysis is a statistical procedure used for evaluating if there is any significant relationship between two unordered categorical variables or not^39,40^. It is based on the Chi-squared test statistic, which measures the difference between the observed frequencies of the two variables and the expected frequencies under the assumption that there is no association. The Chi-squared test statistic can be mathematically formulated as^41,42^:

$$\begin{aligned} \chi ^2 = \sum _{i=1}^r \sum _{j=1}^c \frac{(O_{ij} - E_{ij})^2}{E_{ij}} \end{aligned}$$

In this case, $O_{ij}$ is the observed frequency in a contingency table located in *i*th row and *j*th column, $E_{ij}$ is the expected frequency under the assumption of no association, and the contingency table comprises *r* rows and *c* columns.

If the computed Chi-squared test statistic exceeds the critical value derived from the Chi-squared distribution with $(r-1)\times (c-1)$ degrees of freedom, it leads to the rejection of the null hypothesis. Accordingly, the alternative hypothesis, which indicates a substantial relationship between the variables under study, is accepted. The Chi-squared test is frequently used to determine whether there is a significant relationship between two unordered categorical variables or not. This tool is used for hypothesis testing and can be used in making decisions and policies.

### Gamma test

The gamma test is a statistical technique used to determine if there is any correlation between two ordinal variables or not. Also, the degree of correlation between ordinal variables can be determined by the value of the gamma coefficient^43,44^. The gamma coefficient can be calculated as^45,46^:

$$\begin{aligned} \gamma = \frac{n_{\hbox {concordant}} - n_{\hbox {discordant}}}{n_{\hbox {concordant}} + n_{\hbox {discordant}}} \end{aligned}$$

Among the two variables presented on the right-hand side of the equation, $n_{\hbox {discordant}}$ is the number of discordant observational pairs (the pairs that have different relative orders on the two variables), and $n_{\hbox {concordant}}$ is the number of concordant observational pairs (the pairs that have the same relative order on both variables). Here the denominator represents the total number of observation pairs.

The gamma coefficient ranges from the minimum value of -1 to the maximum value of 1. The value -1 indicates complete disagreement between the two variables, 1 indicates complete agreement and 0 indicates no association. The gamma test is used in a wide variety of fields such as education, psychology, and sociology to measure the relationship between ordinal variables, such as education and income, or job satisfaction and employment length. It is a useful tool for evaluating the strength of association between variables that cannot be measured on a continuous scale.

The chi-squared test is useful in comparing variables if both are equally spaced and the relationship between them is linear. However, this assumption is not satisfied if the data are ordinal in nature. The gamma test does not rely on these assumptions and it can be used to measure the strength of association between ordinal variables that are not linearly related to each other. Therefore, the gamma test may be more appropriate than the chi-squared test for analyzing the relationship between two ordinal variables.

## Results

### Bivariate analyses

Bivariate analysis is one of the simplest statistical approaches for finding an association between two variables. Chi-square and gamma tests were utilized to analyze the relationship between variables and can provide valuable insights into their association. Table 2 indicates that except “Result of salt test”, “High BP” and “Source of drinking water”, all other variables are statistically significant. Among them, the “Highest educational level”, “Wealth index” and “Geographic region” are highly associated. Furthermore, Indian women with improved toilet facilities have a lower risk of maternal anemia.

**Table 2:** Significance of the variable using Chi-square and Gamma test.

Variables	Measurement scale	Chi-square analysis		Gamma test analysis	
		Significance level (*p*-value)	Cramer’s V	Significance level (*p*-value)	Gamma coefficient
Place of residence	Nominal	**0.000**	0.067		
Sex of household head	Nominal	**0.001**	0.022		
Wealth index	Ordinal			**0.000**	0.145
Highest educational level	Ordinal			**0.000**	0.167
Result of salt test	Nominal	0.125	0.012		
High BP	Nominal	0.583	0.006		
Type of toilet facility	Nominal	**0.000**	0.073		
Source of drinking water	Nominal	0.546	0.007		
Geographic region	Nominal	**0.000**	0.096		
Religion	Nominal	**0.000**	0.069		
BMI	Nominal	**0.000**	0.036		
Age	Nominal	**0.000**	0.037		

Significant values are in [bold].

### Multivariate analysis

multivariate analysis allows for the analysis of the relationship between a categorical dependent variable and multiple independent variables. As discussed, both ordinal and multinominal logistic regression is used for multivariate analysis. Features such as the source of drinking water, High BP, and Salt test results were suggested as insignificant by both bivariate and multivariate analyses. Hence, we did not discuss these features in these sections. Several noteworthy observations were made during these analyses, which are briefly discussed in the following sections.

#### Ordinal logistic regression

Ordinal logistic regression studies the ordered nature of the target variable, that is the severity of anemia. Table 3 shows the findings of ordinal logistic regression. This analysis found relationships between several critical factors and anemia in detail. The observational outcome is highlighted below:

- Pregnancy-associated anemia is more common in urban India than rural areas. Urban pregnant Indians are 9.4% less susceptible to severe to moderate anemia than their rural counterparts(OR = 1.094, *p* = 0.007).
- Socioeconomic status significantly influences the prevalence of pregnancy-associated anemia in India. Where the poorest individuals have 34.2% more likelihood of being affected by severe to moderate anemia than the richest ones (OR = 0.658, *p*< 0.001), the prevalence of it reduces as the wealth index improves to poorer(OR = 0.768, *p*< 0.001), middle(OR = 0.833, *p*< 0.001) and richer(OR = 0.846, *p*< 0.001).
- Educated women are observed to have more resistance to the prevalence of anemia. When considering higher education as the reference level, pregnant women with minimum or no education were 37%(OR = 0.621, *p*< 0.001), with primary education were 32% (OR = 0.675, *p*< 0.001) and those with secondary education were 22.2% (OR = 0.778, *p*< 0.001) more vulnerable to being affected by anemia. It is observed that the higher the education level of the individual, the lower the odds of being anemic.
- Type of toilet facility is another concerning factor of anemia in Indian pregnants. Women using improved toilet facilities have a 7.5% less probability of being severe to moderately anemic (OR = 1.075, *p* = 0.032).
- For analyzing anemia prevalence across the geographic regions, Southern regions of India have been considered as the reference category. The Northeastern and Northern regions have a comparatively lower likelihood of severe to moderate anemia (OR = 1.359, *p*< 0.001; OR = 1.130, *p* = 0.003), whereas Eastern parts of India have 17.4% more prevalence of anemia than the southern parts (OR = 0.826 *p*< 0.001).
- The spread of anemia is different among religious groups. Christian pregnants have the lowest odds of being anemic, that is 31.2% (OR = 1.688. *p*< 0.001), and Muslim pregnant women have a 74.5% (OR = 1.255, *p*< 0.001) probability of being anemic when compared with the baseline group.
- Underweight pregnant mothers are found to have the highest likelihood of anemia (OR = 0.993, *p* = 0.914). Moreover, the odds of being anemic decrease as the BMI increases when the obese group is considered as the reference category. Pregnant women of healthy weight have 3.1% (OR = 1.031, *p* = 0.618), and overweight pregnant women have 6.6% (OR = 1.066, *p* = 0.328) fewer odds of being anemic respectively.
- Age of the pregnant women is another concerning factor of pregnancy-associated anemia. While teenage pregnants have the highest probability of being anemic (OR = 0.951, *p* = 0.742), the rate decreases as the age of pregnant women increases.

**Table 3:** Estimations of the parameters of correlated predictor variables using Ordinal Logistic Regression model.

Features	Categories	Estimate (E)	Odds Ratio (OR)	Significance level (*p*-value)	95% C.I. of E	
					L.B.	U.B.
Place of residence	Urban	0.090	1.094	**0.007**	0.025	0.156
	Rural					
Sex of household head	Male	0.055	1.057	0.087	− .008	0.119
	Female					
Wealth index	Poorest	− .419	0.658	**0.000**	− .523	− .315
	Poorer	− .264	0.768	**0.000**	− .359	− .170
	Middle	− .183	0.833	**0.000**	− .272	− .094
	Richer	− .167	0.846	**0.000**	− .253	− .081
	Richest					
Highest educational level	No education, preschool	− .476	0.621	**0.000**	− .568	− .383
	Primary	− .392	0.675	**0.000**	− .488	− .296
	Secondary	− .251	0.778	**0.000**	− .322	− .180
	Higher					
Result of salt test	No iodine	0.044	1.045	0.386	− .056	0.144
	Iodine present					
High BP	No	0.041	1.042	0.399	− .054	0.136
	Yes					
Type of toilet facility	Improved	0.072	1.075	**0.032**	0.006	0.138
	Unimproved	− .044	0.957	0.469	− .162	0.075
	No facility					
Source of drinking water	Improved	− .027	0.974	0.462	− .097	0.044
	Unimproved					
Geographic region	Northern	0.122	1.130	**0.003**	0.041	0.204
	Western	− .029	0.971	0.526	− .118	0.061
	Eastern	− .191	0.826	**0.000**	− .279	− .102
	Northeastern	0.307	1.359	**0.000**	0.202	0.413
	Central	0.058	1.060	0.250	− .041	0.157
	Southern					
Religion	Hindu	0.095	1.100	0.109	− .021	0.212
	Muslim	0.227	1.255	**0.000**	0.102	0.352
	Christian	0.523	1.688	**0.000**	0.381	0.665
	Other/no religion					
BMI	18.5 (underweight)	− .007	0.993	0.914	− .142	0.127
	18.5 to 24.9 (Normal weight)	0.030	1.031	0.618	− .089	0.149
	25 to 29.9 (Overweight)	0.063	1.066	0.328	− .064	0.191
	30 or more Obese					
Age	< = 20	− .050	0.951	0.742	− .351	0.250
	21 to 30	− .004	0.996	0.980	− .300	0.293
	31 to 40	0.070	1.072	0.649	− .231	0.371
	40+					

Significant values are in [bold].

#### Multinominal logistic regression

Multinominal logistic regression considers the target variable as having a set of unordered categories defining different degrees of anemia. Table 4 summarizes the outcome of multivariate logistic regression, which illustrates the relationship between the estimated parameters of the covariates used. The key findings are pointed out below:

- Table 4 reveals a statistically significant difference in anemia prevalence between rural and urban pregnant women, with urban women exhibiting 9.5% lower odds of being severe to moderately anemic (OR = 0.905, *p* = 0.021) and 9.1% lower odds of being mild anemic (OR = 0.909, *p* = 0.029) compared to rural Indian pregnants.
- The type of family structure (patriarchal or matriarchal) did not have any significant impact on the prevalence of severe to moderate anemia, but it has an influence on the prevalence of mild anemia. Indian women of patriarchal families are 12.5% less susceptible to maternal anemia than ones belonging to a matriarchal family.
- Socioeconomic status highly influences the prevalence of anemia. Severe to moderate anemia is more likely to occur in pregnant females from lower socioeconomic backgrounds. Compared to pregnant women of the richest families, severe to moderate anemia is more common in the poorest pregnant women by 69.8% (OR = 1.698, *p*< 0.001). Poorer, middle and richer pregnant women had 39.5%, 27.2%, and 24.7% more likelihood of severe to moderate anemia respectively (OR = 1.395, *p*< 0.001; OR = 1.272, *p*< 0.001; OR = 1.247, *p*< 0.001). Mild anemia is also 33.3% and 17.9% more prevalent in the poorest and poorer pregnant women compared to the richest group.
- Educational status also has a significant impact on anemia prevalence. Compared to educated pregnant, it is found that lower levels of educational qualification are associated with higher odds of being anemic for both severe to moderate(OR = 1.646, *p*< 0.001; OR = 1.376, *p* < 0.001 ) and mild anemic (OR = 1.272, *p*< 0.001; OR = 1.182, *p*< 0.001) cases while considering “higher” as the baseline category. The absence of formal education (“no education”, OR = 1.836, *p*< 0.001; OR = 1.316, *p*< 0.001) is correlated with an increased probability of both severe to moderate and mild anemia. It is observed that increased levels of education decrease the likelihood of anemia.
- The type of toilet system used also plays an important role in anemia prevalence. The pregnants using safer sanitation systems are found to be 9.2% less vulnerable to severe to moderate anemia than the users of no proper toilet facilities (OR = 0.908, *p* = 0.024).
- The prevalence of anemia varies across geographical locations. Northeastern regions have 30.4% (OR = 0.696, *p*< 0.001) and 21.2% (OR = 0.788, *p* = 0.001) less likelihood of severe to moderate and mild maternal anemia when considering the Southern region as the reference category. In Northern states of India, the probability of severe to moderate anemia is 11.2% (OR = 0.888, *p* = 0.027) and mild anemia is 16.4% (0.836, *p* = 0.001) lower than in Southern India. On the other hand, pregnant women residing in eastern India are 26.8% more prone to severe to moderate anemia (OR = 1.268, *p*< 0.001) and 29.6% more prone to mild anemia (OR = 1.296, *p*< 0.001) compared to the baseline category.
- Investigating the influence of religious affiliation on anemia severity, the findings exposed intriguing patterns. Muslim pregnant women exhibited a reduced likelihood of 26.5% severe to moderate anemia(OR = 0.735, *p*< 0.001). Conversely, Christian women displayed a 48.1% lower probability for severe to moderate anemia (OR = 0.519, *p*< 0.001), and a 27.3% lower probability of mild anemia (OR = 0.727, *p* = 0.001) with respect to “other/no religion” group as reference.
- Maternal anemia is associated with the age of the individuals as well. Especially in the case of mild anemia, teen Indian mothers are 67.6% more vulnerable to mild anemia (OR = 1.676, *p* = 0.027) than pregnants in/above their forties. Also, pregnants in their twenties have 56.7% more likelihood of experiencing mild anemia (OR = 1.567, *p* = 0.51)than pregnant individuals aged forty and above.
- The probability of being both severe to moderate and mild anemic is more pronounced in pregnants with lower BMI. As the BMI increases, their likelihood of being anemic decreases. The ‘obese’ group has been considered as the reference category.

**Table 4:** Estimations of the parameters of correlated predictor variables using Multinominal Logistic Regression model.

Features	Categories	Odds Ratio (OR)		Significance level (*p*-value)		95% C.I. of E			
						LB		UB	
		Severe to Moderate vs not anemic	Mild vs Not Anemic	Severe to Moderate vs Not Anemic	Mild vs Not Anemic	Severe to Moderate vs Not Anemic	Mild vs Not Anemic	Severe to Moderate vs Not Anemic	Mild vs Not Anemic
Place of residence	Urban	0.905	0.909	**0.021**	**0.029**	0.831	0.834	0.985	0.990
	Rural	.	.	.	.	.	.	.	.
Sex of household head	Male	0.953	0.875	0.246	**0.002**	0.878	0.806	1.034	0.951
	Female	.	.	.	.	.	.	.	.
Wealth index	Poorest	1.698	1.333	**0.000**	** 0.000**	1.484	1.163	1.945	1.529
	Poorer	1.395	1.179	**0.000**	**0.009**	1.234	1.043	1.578	1.334
	Middle	1.272	1.084	**0.000**	0.177	1.132	0.964	1.430	1.217
	Richer	1.247	1.083	**0.000**	0.161	1.114	0.969	1.396	1.211
	Richest	.	.	.	.	.	.	.	.
Highest educational level	No education, preschool	1.836	1.316	**0.000**	**0.000**	1.629	1.164	2.070	1.488
	Primary	1.646	1.272	**0.000**	**0.000**	1.454	1.121	1.864	1.444
	Secondary	1.376	1.182	**0.000**	**0.000**	1.253	1.079	1.511	1.296
	Higher	.	.	.	.	.	.	.	.
Result of salt test	No iodine	0.940	0.986	0.345	0.832	0.828	0.864	1.068	1.125
	Iodine present	.	.	.	.	.	.	.	.
High BP	No	0.948	0.980	0.387	0.753	0.839	0.863	1.070	1.112
	Yes	.	.	.	.	.	.	.	.
Type of toilet facility	Improved	0.908	0.949	**0.024**	0.250	0.835	0.869	0.987	1.037
	Unimproved	1.046	1.030	0.561	0.716	0.899	0.879	1.216	1.207
	No facility	.	.	.	.	.	.	.	.
Source of drinking water	Improved	1.037	1.013	0.433	0.780	0.947	0.923	1.136	1.112
	Unimproved	.	.	.	.	.	.	.	.
Geographic regions	Northern	0.888	0.836	**0.027**	**0.001**	0.799	0.751	0.987	0.930
	Western	1.059	0.952	0.328	0.420	0.944	0.846	1.188	1.072
	Eastern	1.268	1.296	**0.000**	**0.000**	1.130	1.154	1.422	1.457
	Northeastern	0.696	0.788	**0.000**	**0.001**	0.607	0.686	0.798	0.904
	Central	0.925	1.024	0.235	0.721	0.814	0.900	1.052	1.165
	Southern	.	.	.	.	.	.	.	.
Religion	Hindu	0.866	1.056	**0.054**	0.501	0.747	0.901	1.002	1.238
	Muslim	0.735	0.964	**0.000**	0.674	0.628	0.813	0.861	1.143
	Christian	0.519	0.727	**0.000**	**0.001**	0.432	0.601	0.624	0.879
	Other/no religion	.	.	.	.	.	.	.	.
BMI	18.5 (underweight)	1.018	1.018	0.838	0.842	0.855	0.853	1.213	1.214
	18.5 to 24.9 (Normal weight)	0.981	0.940	0.804	0.437	0.840	0.805	1.145	1.098
	25 to 29.9 (Overweight)	0.947	0.907	0.516	0.248	0.802	0.768	1.117	1.071
	30 or more Obese	.	.	.	.	.	.	.	.
Age	< = 20	0.975	1.676	0.893	**0.027**	0.678	1.062	1.403	2.646
	21 to 30	0.927	1.567	0.679	**0.051**	0.648	0.997	1.327	2.462
	31 to 40	0.855	1.432	0.399	0.124	0.593	0.906	1.231	2.262
	40+								

Significant values are in [bold].

## Discussion

The study identifies several important factors that increase the probability of pregnancy anemia in India. The study indicates that the prevalence of anemia during pregnancy is possible to mitigate by designing a targeted scheme based on the analysis of data about various variables such as place of residency location, the origin of potable water, type of toilet structure, sex of the head of the family, wealth index, the religious affiliation of the head of household, educational level, geographic region, iodine level in salt, and high blood pressure.

The findings of this study indicate that pregnant women who reside in rural areas exhibit a higher likelihood of developing anemia than their urban counterparts. The results of the study are similar to those of several other studies^47,48^. The pace of modernization and industrialization in rural areas is slower than in urban areas. Health services are often unavailable to rural communities, and pregnant women are mostly unaware of proper nutrition, which could contribute to higher rates of anemia. In order to reduce the prevalence of anemia in India, effective interventions and government programs should be designed based on the disparities in socioeconomic, health, and nutrition factors between rural and urban areas.

This study clearly indicates that education plays a vital role in preventing pregnancy-associated anemia. One who receives education possesses better nutrition knowledge and a better understanding of pregnancy factors compared to someone who is not privileged to have access to education. By being aware and knowledgeable, educated people can better take care of themselves or other women during pregnancy and reduce their risk of developing anemia^49,27^. The literacy rate among women is one of the most crucial underlying factors of the prevalence of anemia. An educated woman typically has more access to healthcare services and facilities, which helps reduce the risk of anemia. With the increasing level of education, women become more equipped with knowledge of the health risks associated with anemia. Consequently, the more the women are educated the better they are able to prevent or manage the condition, reducing its chances of occurring.

This study revealed that, in India, pregnancy-associated anemia and affiliation to religious groups have a significant association, which resonates with several other studies^50,51^. According to the findings of our research, pregnant belonging to Hindu communities suffer more from anemia compared to other religious groups. Pregnancy-associated anemia was less prominent in the Christian community. This variation in the prevalence of anemia between different religious groups may be because of the differences in their dietary habits and lifestyles.

A significant association was found between the socioeconomic status of pregnant women and their risk of anemia. Pregnant women from low-income households in India are more susceptible to anemia than their high-income counterparts. Earlier research studies also testify the same findings^27,52^. This could be due to a lack of access to quality healthcare services. Poorer families may also have limited access to a wide range of foods, which can result in poor nutrition and anemia. Therefore, there is a crucial need for targeted efforts to address the issue of anemia in pregnant women from low-income families.

This research found an inverse relationship between the availability of toilet facilities and the occurrence of pregnancy-associated anemia. Not only this research, but some other studies also support the same findings^53,54^. If empirically analyzed, possible explanations for this relationship can be found. Improved toilet facilities help to reduce many parasitic infections, which are common causes of anemia, for example, hookworm. Additionally, improved sanitation facilities can promote better hygiene practices, such as handwashing, which can prevent other infectious diseases that may later cause anemia. The findings indicate how ensuring basic needs such as access to safe and hygienic sanitation facilities can have a greater influence on improving maternal health. It also suggests that investing in sanitation infrastructure could have a significant impact on reducing the burden of anemia among pregnant women.

As observed from the findings, the prevalence of anemia is not the same across all the regions in India, demonstrating the importance of dedicated interventions to tackle the problem. In this case, also, various other studies also validate the same finding^55,56^. eastern regions of India showed high rates of pregnancy-associated anemia, which is quite alarming and requires immediate attention to control and prevent it. Such preventive measures may include increasing access to iron and folic acid supplements, nutrition education, and improving healthcare services for pregnant women in these geographic regions. It is encouraging to note that northern and western India have a significantly lower prevalence of pregnancy-associated anemia compared to other regions. There are plenty of reasons behind this, including better access to healthcare services and nutrition, higher levels of education, etc. in these regions. It can set a valuable example for other Indian geographic regions in implementing effective interventions aimed at reducing maternal anemia.

Prevalence of maternal anemia in India is not even for all age groups, rather specific age groups are more affected. Particularly, mild anemia is more prevalent in teenage and young adult Indian mothers. This finding is evident in several other studies as well^57,58^. It may be due to underlying factors such as inadequate nutrition, limited prenatal care, socioeconomic disparities, and early pregnancies. On the other hand, pregnant women aged over 40 are more susceptible to severe anemia, probably due to reduced ability of nutrient absorption, menopausal changes, chronic illnesses, dietary patterns, and so on.

Resonating with several other studies^59,47^, the probability of pregnancy-associated anemia is higher in underweight women. Lower BMI is associated with malnutrition, indicating inadequate nutritional reserves in the body, such as iron, vitamin B12, and folic acid which are essential for the production of red blood cells. Conversely, individuals with a healthy BMI are more likely to have a balanced and nutritious diet, reducing the risk of nutritional deficiencies leading to anemia.

This research leads to an unexpected finding, that is the absence of a significant impact of iodized salt on pregnancy-associated anemia. Iodine deficiency is a well-known cause of goiter, intellectual impairment, and cretinism. Pregnant women require higher amounts of iodine to ensure proper development of the fetal brain, and therefore iodine supplementation is crucial during pregnancy. Our study suggests that, although iodized salt may help to prevent iodine deficiency, it may not be significantly impactful to the prevention of anemia in pregnant women. However, this result contradicts some of the existing studies^56,60^.

## Limitation and future work

There are some limitations to our study of pregnancy-related anemia in India. Firstly, our reliance on the DHS 2019–2021 dataset restricts our findings to a timeframe, which means we might miss out on some recent changes in anemia prevalence. Moreover, the cross-sectional nature of the data limits our ability to establish causal relationships highlighting the need for longitudinal studies to understand how these dynamics evolve over time. Additionally, the variables available in the dataset limit the scope of our analysis causing some other influential factors of anemia to be left out. It is crucial to include variables, than what the DHS dataset provides in order to encompass all the factors involved. Additionally investigating the efficacy of interventions aimed at addressing these factors could lead to some promising strategies to minimize the occurrence of pregnancy-related anemia. India, with its range of geography, culture, and economy, is a country of diversity. Therefore it would be more beneficial to concentrate on research that explores estimations at the micro level such as districts and PSUs, in order to gain insights for formulating more targeted policy recommendations.

## Conclusion

This investigation aimed to identify the underlying determinants of anemia among pregnant females in India by utilizing information from the Demographic and Health Survey from 2019–21 (India). More than half of the pregnant females in our research sample had anemia, indicating a significant prevalence of anemia in India. Our analysis identified several essential parameters related to suffering from anemia by pregnant females, including the highest educational level, wealth index, residing geographical region, type of toilet structure, the religious affiliation of the household head, urbanicity of the residence, age, BMI, and family structure. From the observations of bivariate and multivariate analyses, the highest educational level, wealth index, and geographical region were identified as the most crucial elements that influence anemia among pregnant women in India. These findings have important implications for policymakers and healthcare providers in India. Interventions targeted toward enhancing antenatal care services can potentially alleviate the prevalence of anemia among pregnant females. Additionally, efforts to improve education and economic opportunities for women may also have a positive impact on the prevention and control of anemia. Overall, this study emphasizes the need for targeted interventions to mitigate the alarming prevalence of anemia among pregnant females in India.

## Data Availability

The datasets used and/or analysed during the current study available from the corresponding author on reasonable request.
